# Simultaneous Optical and Mechanical Sensing Based on Optomechanical Resonators

**DOI:** 10.1021/acssensors.3c02103

**Published:** 2023-12-29

**Authors:** Elena Sentre-Arribas, Alicia Aparicio-Millán, Aristide Lemaître, Ivan Favero, Javier Tamayo, Montserrat Calleja, Eduardo Gil-Santos

**Affiliations:** † OptoMechanicalSensors Lab, Instituto de Micro y Nanotecnología, IMN-CNM (CSIC), Isaac Newton 8 (PTM), E-28760 Tres Cantos, Madrid Spain; ‡ Centre de Nanosciences et de Nanotechnologies, Université Paris-Saclay, CNRS, UMR 9001, 91120 Palaiseau, France; § Matériaux et Phénomènes Quantiques, Université Paris Cité, CNRS, UMR 7162, 75013 Paris, France; ∥ Bionanomechanics Lab, Instituto de Micro y Nanotecnología, IMN-CNM (CSIC), Isaac Newton 8 (PTM), E-28760 Tres Cantos, Madrid Spain

**Keywords:** optomechanical resonators, optical and mechanical sensors, multiparametric sensing, biological and chemical sensing, environmental monitoring

## Abstract

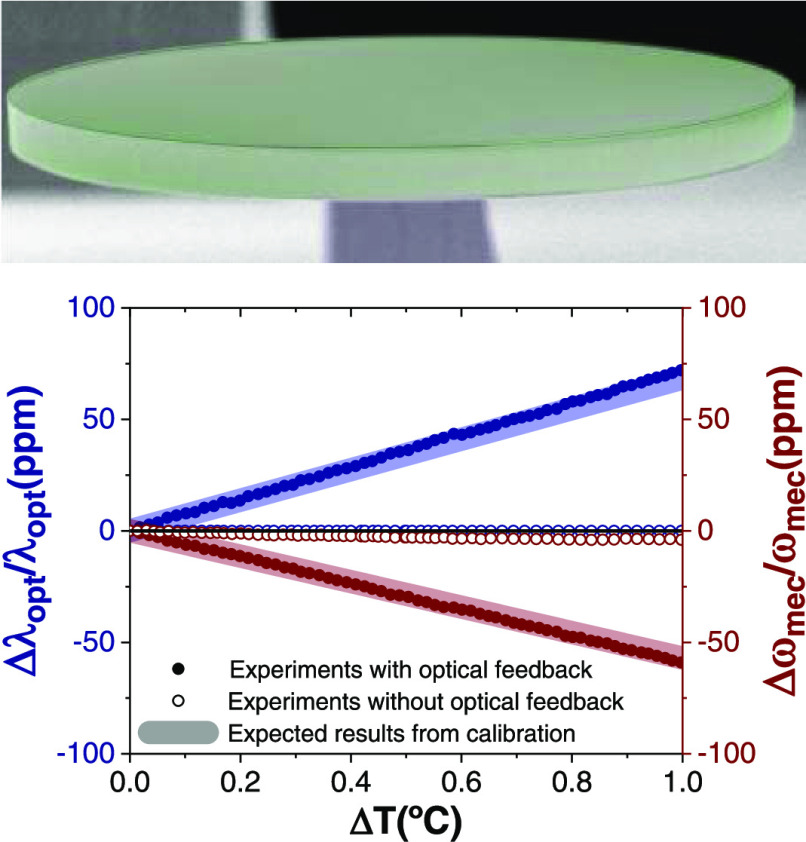

Optical and mechanical resonators have each been abundantly employed in sensing applications, albeit following separate development. Here we show that bringing together optical and mechanical resonances in a unique sensing device significantly improves the sensor performance. To that purpose, we employ nanoscale optomechanical disk resonators that simultaneously support high quality optical and mechanical modes localized in tiny volumes, which provide extraordinary sensitivities. We perform environmental sensing, but the conclusions of our work extend to other sensing applications. First, we determine optical and mechanical responsivities to temperature and relative humidity changes. Second, by characterizing mechanical and optical frequency stabilities, we determine the corresponding limits of detection. Mechanical modes appear more sensitive to relative humidity changes, while optical modes appear more sensitive to temperature ones, reaching, respectively, 0.05% and 0.6 mK of independent resolution. We then prove that simultaneous optical and mechanical monitoring enables disentangling both effects and demonstrates 0.1% and 1 mK resolution, even considering that both parameters may change at the same time. Finally, we highlight the importance of actively tracking the optical mode when optomechanical sensor devices. Not doing so enforces tedious independent calibration, influences the device sensitivity during the experiment, and shortens the sensing range. The present work hence clarifies the requirements for the optimal operation of optomechanical sensors, which will be of importance for chemical and biological sensing.

In the last decades, an assortment of optical and mechanical resonators has been successfully tested in a variety of sensing applications such as environmental monitoring, industrial chemical processing, and biomedicine, among many others.^1−7^ Importantly, the responsivity of both optical and mechanical platforms usually increases by reducing the devices size.^8^ The rapid advancement in micro- and nanofabrication technologies has accelerated this miniaturization, significantly improving their sensitivities. Nonetheless, it is worth emphasizing that the sensor performance is also set by its capability to discern between unspecific and specific signals. Implementing efficient and robust sensors does not restrict the provision of very high sensitivity but also, and even more important, very high reliability. While this is true for every sensing application, it is of particular importance for biological/chemical sensing. In this regard, researchers proposed characterizing the intrinsic physical properties of the detected analytes individually, which may enable their univocal identification.^9^ As an additional advantage, this method does not require functionalizing the sensor surface, allowing the sensing of many different analytes in a single device.

Both platforms stand out for being extraordinarily sensitive, even if they present advantages and disadvantages depending on the particular application. Regarding biological/chemical sensing, mechanical resonators attracted attention due to their capability of accurately characterizing the mechanical properties, mass, and shape of the detected analytes, potentially leading to their unambiguous identification.^10,11^ With these devices, researchers have successfully detected, characterized, and identified individual human cells, bacteria, viruses, and even proteins.^12−15^ Unfortunately, most of these achievements were attained under high-vacuum conditions while operating the devices in a liquid environment has remained difficult. Mechanical resonators generally suffer high energy losses when immersed in liquids, which dramatically hampers their assets.^16,17^ Importantly, optical resonators do not present this drawback as their capabilities remain almost unaltered when operating in fluids. With optical resonators immersed in liquid, researchers succeeded in the detection of a single virus.^18^ Regrettably, the optical properties of the analytes are less deterministic than the mechanical properties, precluding their unequivocal identification.

More recently, optomechanical resonators have emerged as a promising platform for different sensing applications, going from monitoring chemical and biological processes to characterizing surfaces and fluids, through the detection of very small forces.^19−29^ Importantly, optomechanical effects enable access to particular mechanical modes that present outstanding characteristics. These modes are localized in very small volumes and resonate at a very high frequency, leading to sensitive and fast sensing capabilities. Several mechanical modes employed in optomechanics present an in-plane nature, implying moderate mechanical dissipation when immersed in fluids, which makes them interesting candidates for in liquid applications.^19^ Combining these assets with the concomitant use of the optical modes supported by optomechanical devices may provide complementary sensing information, an avenue that remains to be explored.^29^ In addition, it may also help to distinguish unspecific signals from specific signals. Such an approach increases the sensors capabilities and, notably, their reliability. In biological and chemical sensing, this method may enable simultaneous access to the optical and mechanical properties of the analyte, providing more complete characterization and recognition.

In this work, we highlight this potential by using devices that simultaneously support very high quality optical and mechanical resonances, both confined in tiny volumes (<μm^3^). Bringing together optical and mechanical modes in a unique sensing platform allows harnessing the assets of both approaches while reducing their limitations. In particular, we demonstrate the advantages of the dual sensing approach by applying nano-optomechanical disks for monitoring environmental conditions. We first characterize their optical and mechanical responses to temperature and relative humidity changes. We then analyze the stability of their optical and mechanical resonant frequencies as a function of the injected optical power. Finally, we reach a resolution of determining temperature and relative humidity changes of 1 mK and 0.1%, respectively. Importantly, we demonstrate it even when both parameters change at the same time, as simultaneously monitoring the optical and mechanical resonant frequencies enables disentangling temperature and relative humidity changes. Lastly, we study the impact of varying optical detuning on the mechanical sensing response. We conclude that actively tracking the optical mode is essential to obtaining reliable mechanical signals, avoiding tedious post-treatment and extra calibration.

## Experimental Setup

**Figure 1:**
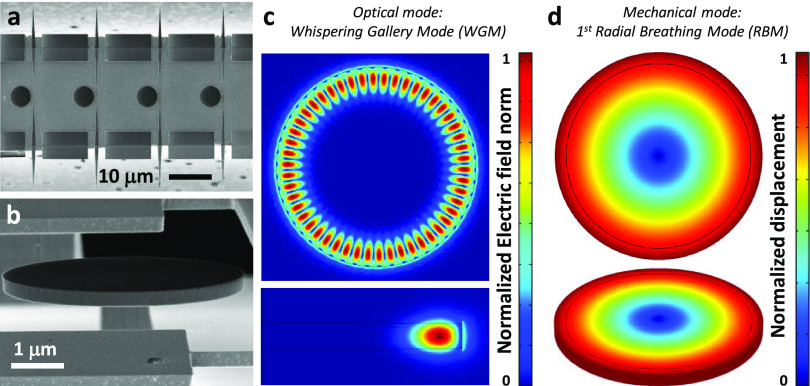
Nano-optomechanical disks. (a) Scanning electron microscopy (SEM) image of a series of nano-optomechanical GaAs disks (R = 2.5 μm, *t* = 320 nm), observed from top. The image also shows the fully suspended optical waveguides, which enable evanescently coupling light into the disks. (b) SEM side view of a nano-optomechanical disk tilted at an angle of 80°. The image also shows the AlGaAs pedestal on which the disk rests. (c) Top and side views of the electric field distribution associated with the TE_1,25,1_ Whispering Gallery Mode (WGM) of the disk, obtained through Finite Element Method (FEM) simulations. The color scale represents the normalized electric field norm. (d) Top and tilted views of the displacement field associated with the 1st RBM of the disk, obtained through FEM simulations. The color scale represents the normalized displacement.

Arrays of nano-optomechanical disks with on-chip integrated optical waveguides were fabricated out of Gallium Arsenide/Aluminum Gallium Arsenide (GaAs/AlGaAs) wafer (Figure 1a). The GaAs disks are 320 nm in thickness and 2.5 μm in radius, and they rest on AlGaAs pedestals, which are 200–500 nm in radius and 1.8 μm in height (Figure 1b). The integrated waveguides were designed in order to maximize the optical laser light injection, collection, and transmission. They are fully suspended, and tapered at both endings, reaching a total optical transmission of up to 30%. In order to optimize evanescent coupling to the disk modes, they are 180 nm in width in the vicinity of the disks, and the gap distance to the disk is 340 nm. Nano-optomechanical disks support both optical and mechanical modes, which present key assets.^30^ Optically, they support the Whispering Gallery Modes (WGMs), where light circulates around their periphery by total internal reflection. Figure 1c illustrates the normalized electric field distribution of the particular WGM used in this work, labeled as *TE*_1,25,1_, whose main component of the electric field is in the disk plane.^31^ The *p*, *m*, and *l* subscripts correspond to the number of lobes displayed by the mode in the radial, azimuthal, and vertical directions, respectively. WGMs in GaAs disks hold remarkably low energy dissipation, reaching optical quality factors of over10^6^, which provides them with a very high sensitivity. They strongly couple to the family of mechanical Radial Breathing Modes (RBMs), wherein the disk expands and contracts radially. Figure 1d shows the normalized displacement of the first RBM, which was employed along this work. Thanks to the very high optomechanical coupling present in these devices (*g*_om_ ≈ 500 GHz/nm),^32^ it is possible to reach a displacement sensitivity of around 10^–18^ m/√Hz, which allows resolving the RBM Brownian vibrations with large signal-to-noise. RBMs resonate at extremely high frequencies, up to the GHz range, and exhibit moderate energy dissipation when immersed in fluids, which provides them with extremely high mechanical sensitivity and speed in the detection of signals.

**Figure 2:**
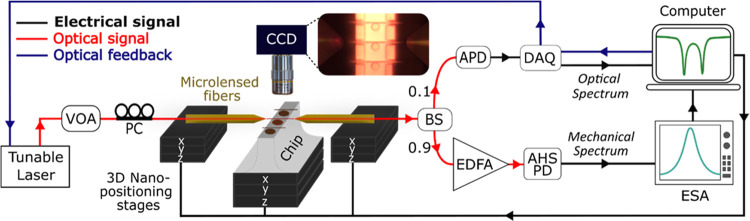
Schematics of the optomechanical setup used in this work. Light from a near-infrared tunable laser is polarization and power controlled, through a polarization controller (PC) and a variable optical attenuator (VOA), before being injected in and collected from the devices using microlensed fibers. The microlensed fibers and the chip containing the devices are mounted on three-dimensional nanopositioning stages, which together with a homemade visualization system enable their proper alignment. The collected light is split (90:10) using a fiber beam splitter (BS). 10% of light is sent onto an amplified photodetector (APD) connected to a data acquisition card (DAQ). The remaining 90% is sent through an Erbium-Doped Fiber Amplifier (EDFA), whose output is connected in series to an Amplified High-Speed Photodetector (AHSPD) and an electrical spectrum analyzer (ESA). The DAQ signal enables implementing a feedback loop, which acts on the laser optical wavelength to keep the optical detuning to the WGM resonance constant. The inset shows an optical image, taken with the homemade visualization system, of a series of nano-optomechanical disks, their integrated on-chip waveguides, and the microlensed fibers.

The optomechanical setup used in this work (Figure 2) allows characterizing several WGMs and RBMs of the nano-optomechanical disks. Importantly, it enables monitoring the resonant frequency associated with a WGM and a RBM of a disk with very high resolution, in real time, and simultaneously. Light from a near-infrared tunable laser (T100S-HP, EXFO Inc.) is polarization and power controlled, before injecting and collecting it into/from the integrated optical waveguides using microlensed fibers (TSMJ-3A-1550, AMS Technologies AG). The microlensed fibers and the chip containing the devices are mounted on three-dimensional nanopositioning stages (MAX311D/M, Thorlabs Inc.), which together with a homemade optical microscope enable their precise alignment. The collected light is split into two branches. 10% of the collected light is directly sent onto an amplified photodetector (PDA10CS2, Thorlabs Inc.), which is connected to a data acquisition card (BNC-2110, National Instruments Corp.). Primarily, this signal serves for measuring the disk optical transmission spectra, enabling the characterization of their WGMs. After selecting a particular WGM, attending to its contrast, quality factor, and optomechanical coupling, the laser′s optical wavelength is set on its blue flank. As a result, the collected light gathers information about the disk mechanical vibrations. To access this information, the remaining 90% of the collected light is amplified using an erbium-doped fiber amplifier (CEFA-C-HG, Keopsys by Lumibird), and then sent onto an amplified high-speed photodetector (DXM20AF, Thorlabs Inc.). The photodetector′s electrical output is finally inspected using an electrical spectrum analyzer (FSV7, Rohde& Schwarz GmbH& Co KG), which provides access to the disk thermomechanical spectrum and, in particular, to the Brownian motion spectrum of the first RBM.

The devices are placed inside an environmentally controlled chamber, which is equipped with a thermocouple (TFC-305P, CIE-Group Ltd.) and a relative humidity gauge (Testo 635, Testo SE& Co. KGaA). It possesses two gas inlets, one connected to a nitrogen valve, and the other to a humidifier. By regulating the gas fluxes, we are able to establish the relative humidity inside the chamber with a precision of 1%. Additionally, the chip containing the sensors is mounted on top of a Peltier heater, which allows temperature tuning with an accuracy of 10 mK. Importantly, a dedicated software enables the automatic alignment of the microlensed fibers and the waveguides at the desired time rate, while the environmental conditions change. Furthermore, it enables characterizing the evolution of the optical and mechanical resonant frequency in real time. Optically, it continuously monitors the optical transmission of the system. Note that, when the optical laser wavelength is set on a WGM flank, the optical transmission is related to the WGM resonant wavelength, which is this way tracked. In addition, the software enables activating a feedback loop to keep the optical detuning to the WGM constant. To this end, it maintains the optical transmission of the system, finely tuning the optical laser wavelength. Mechanically, the software continuously fits the acquired mechanical resonance to a damped harmonic oscillator, which provides very accurate information about its mechanical frequency and quality factor. Concretely, we analyze the resonance associated with the disk first RBM. Note that the following results were obtained using the close loop operation mode, meaning that the optical detuning was maintained constant. In the last section of the article, we compare the close loop and open loop operation modes, and highlight the importance of keeping the optical detuning constant during sensing experiments with optomechanical devices.

## Results and Discussion

**Figure 3:**
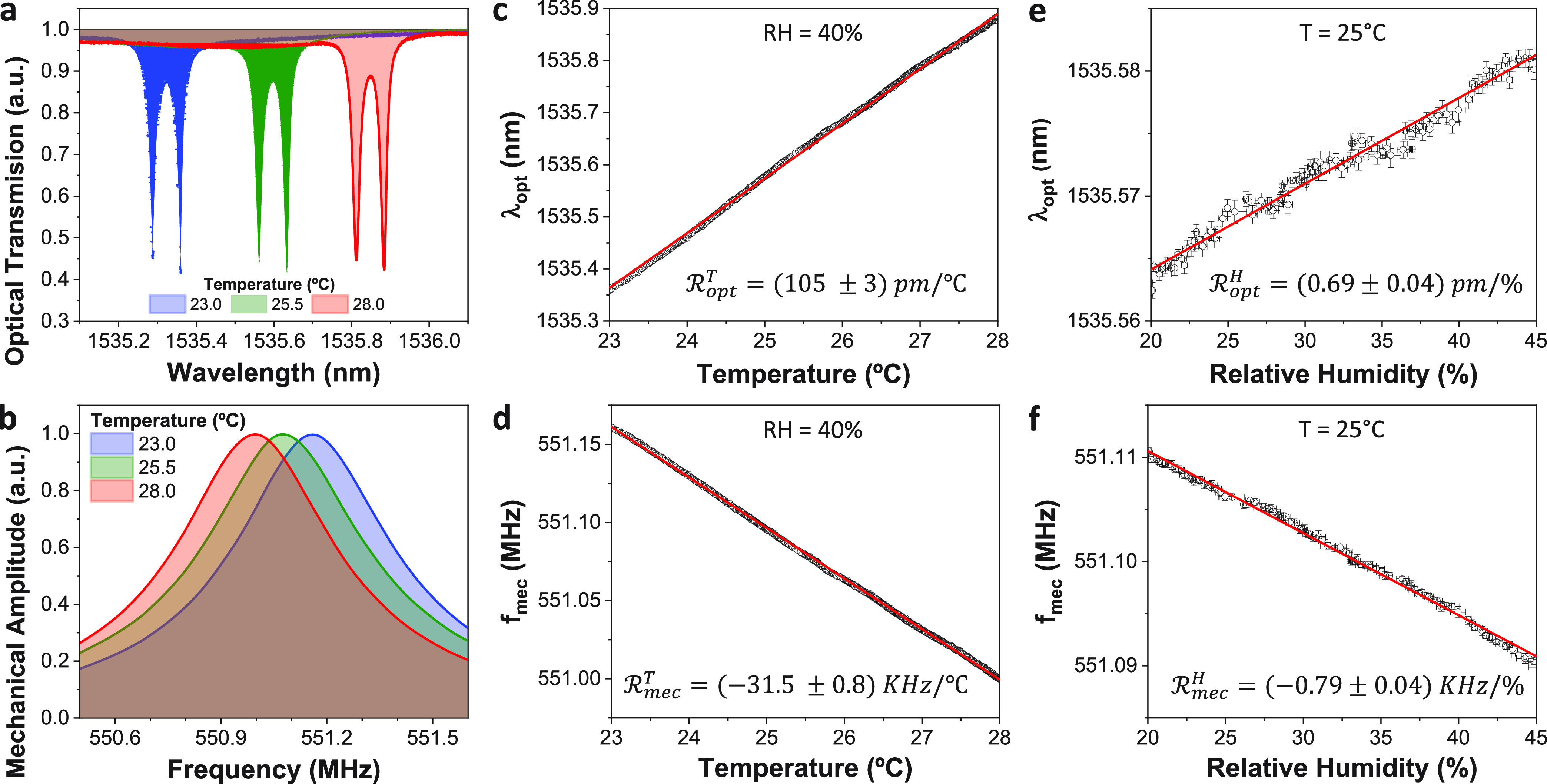
Optical and mechanical responsivity to temperature and relative humidity changes. (a) Optical spectra showing the transmission dips corresponding to the doublet associated with the TE_1,25,1_ WGM of the disk shown in Figure 1b at three different temperatures and at constant relative humidity (40%). (b) Mechanical spectra showing the 1st RBM resonance of the disk, at three different temperatures and at constant relative humidity (40%). (c) Evolution of the disk WGM resonant wavelength and (d) the disk 1st RBM resonant frequency as a function of temperature, at constant relative humidity (40%). (e) Evolution of the disk WGM resonant wavelength and (f) the disk 1st RBM resonant frequency as a function of the relative humidity at constant temperature (25 °C).

To evaluate the assets of our devices as environmental sensors, we first fixed the chamber’s relative humidity to 40% and varied the temperature of the device. Figure 3a,b shows the optical and mechanical spectra of a disk, acquired at three particular temperatures: 23 °C, 25.5, and 28 °C. In these experiments, the optical power at the input microlensed fiber was set to 10 μW, in order to minimize optomechanical effects. Note that, in this regime, we estimate that light absorption increases the disk temperature by less than 200 mK. The optical spectra exhibit the dips corresponding to the doublet associated with the *TE*_1,25,1_ WGM of the disk (Figure 1c). This doublet spectral signature results from the coupling of the clockwise and counterclockwise modes.^33^ The resonant wavelengths and loaded optical quality factors are about 1535.5 nm and 85.000, respectively. On the mechanical side, the spectra show the thermomechanical motion associated with the disk first RBM, whose resonant frequency and quality factor are about 551 MHz and 1300, respectively. Figure 3c,d displays the detailed evolution of the optical and mechanical resonant wavelength and frequency as a function of temperature. The curves bring into light clear linear dependences. As the temperature increases, the WGM resonant wavelength increases, while the RBM resonant frequency decreases. By fitting the experimental data to linear curves, the resulting device responsivities are  and .

Let us gain insight into the physical origin of the measured responsivities and compare them with the expected values. Regarding the optics, the resonant wavelength λ_m_ associated with a WGM of order *m* can be expressed as
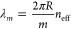
1where *R* is the disk radius, *m* is the azimuthal number, and *n*_eff_ is an effective refractive index. Note that we simplified the notation by eluding the *p* and *l* indexes. Variations in temperature induce changes on both the disk radius and effective refractive index, the latter being the dominant effect. As the temperature increases, the effective refractive index increases too, shifting the optical resonant wavelengths to larger values. Taking into account the reported dependence of the GaAs refractive index with temperature,^34^ together with eq 1, we obtain . Regarding the mechanical responsivity, let us first consider that the disks are made out of an isotropic material. In this case, the RBM resonant frequencies are given by
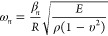
2where *E*, ρ, and υ are the disk Young′s modulus, density, and Poisson ratio, respectively, while β_*n*_ is the eigenvalue associated with the *n*th mode, which takes the value 2.055 for the first RBM. Temperature variations induce changes on the disk radius, density, and Young’s modulus. In this case, the dominant effect comes from the temperature dependence of the Young’s modulus. Therefore, as the temperature rises, since the GaAs Young’s modulus decreases, the mechanical resonant frequency diminishes. Actually, GaAs is an anisotropic material, which transforms eq 2 in a much more complex equation that relates the RBMs frequency to the different elastic coefficients of GaAs. Taking into account the reported variation of the GaAs elastic coefficients with temperature,^35^ we obtain . Note that the experimental responsivities measured for both the WGM and the RBM are in very close agreement with expectations from the materials properties.

In the following experiment, we fixed the device temperature at 25 °C while varying the relative humidity inside the chamber. Figure 3e,f displays the WGM resonant wavelength and the RBM resonant frequency, respectively, as a function of the relative humidity. Just like for temperature changes, both resonances respond in a linear manner for the considered range (relative humidity going from 20 to 45%). Note that as the relative humidity increases, a larger number of water molecules are adsorbed onto the disk surface. Optics wise, it leads to an increase of the disk effective refractive index, hence increasing the WGM resonant wavelength (eq 1). Mechanics wise, the adsorption of water molecules increases the disk effective rigidity and mass, being the latter the dominant effect, which finally decreases the RBM resonant frequency (eq 2).^36,37^ Again, by fitting the experimental data to linear curves, we calculate the optical and mechanical responsivities to relative humidity changes, obtaining:  and . Moreover, by combining the experimental data with FEM simulations, we estimate that (40.6 ± 3.1)% of a water monolayer adsorbs onto the sensor surface when increasing the relative humidity from 20 to 45%. Importantly, the optical and the mechanical results are in excellent agreement, validating this conclusion (Supporting Information Section S1).

**Figure 4:**
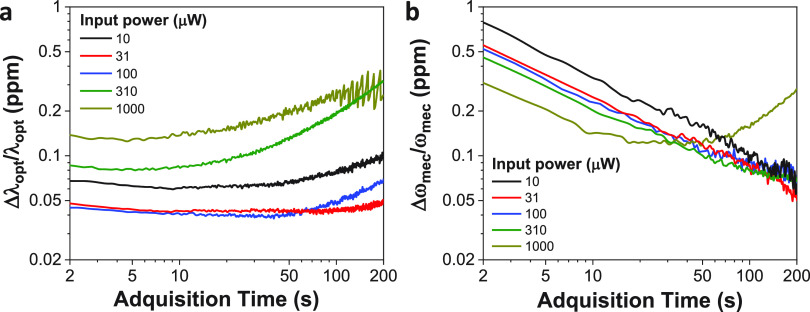
Stability as a function of the optical input power. (a) Allan deviation of the WGM resonant wavelength and (b) Allan deviation of the RBM resonant frequency of the disk shown in Figure 1b, using different optical input powers: 10 μW (black), 31 μW(red), 100 μW (blue), 310 μW(green), and 1 mW (dark yellow).

Let us now analyze the optical wavelength and mechanical frequency stabilities of the proposed device in order to assess its limits in detecting temperature and relative humidity changes. Figure 4a,b shows the Allan deviation for the WGM and RBM, respectively, as a function of the injected optical power. Their behaviors are quite similar. At short acquisition times, increasing the optical input powers enhances the mechanical signal, improving the precision on determining the mechanical resonant frequency. The same occurs for the optical resonant wavelength until surpassing an optical input power of 100 μW. At this point, the resolution starts to decay due to the increase of temperature, which induces thermal instabilities of the WGM. Note that, the mechanical signal does not show this regime because, at short times, its resolution is lower than the optical one. At long acquisition times, the noise level is set by misalignments in between the microlensed fibers and the integrated waveguides, which lead to changes on the sensor temperature. These changes are larger as the optical input power is increased, which decreases the precision attained on determining the WGM resonant wavelength and the RBM resonant frequency. For both cases, the Allan deviation minimum occurs at quite large acquisition times and injects intermediate optical input powers. In particular, using an optical input power of 100 μW, we can determine the WGM resonant wavelength and the RBM resonant frequency with a resolution of 0.04 ppm (ppm) at an acquisition time of 40s, and 0.07 ppm at an acquisition time of 200 s, respectively. Considering the previously measured responsivities, we conclude that WGM sensing provides a lower limit of detection regarding temperature changes than RBM sensing. The contrary occurs when monitoring relative humidity changes, even if, in this case, the mechanical and optical limits of detection are quite similar. Concretely, the minimum detectable change of relative humidity is 0.05%, while the one of temperature is 0.6 mK.

So far, we have considered independent changes in the temperature or relative humidity. However, in most practical situations, both parameters may change at the same time. Considering that temperature and relative humidity effects are additive and linear, which is a good approximation at first order, we deduce from eqs 1–2
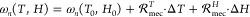
3
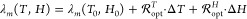
4

Equations 3–4 indicate an important advantage of the dual optical/mechanical sensing approach explored in this work. By individually monitoring the sensor’s WGM resonant wavelength or RBM resonant frequency, it is not possible to distinguish temperature and relative humidity changes. At variance, by simultaneously monitoring optical and mechanical signals, it is possible to disentangle both effects. Note that a necessary condition for disentangling both effects is that the ratio in between the mechanical and the optical responsivities were not identical for both parameters. In particular, considering the optical and mechanical stabilities attained when using an optical input power of 100 μW and an acquisition time of 50 s (Figure 4), together with eqs 3–4, we demonstrate a detection limit of 0.1% in relative humidity change and 1 mK in temperature change. Remarkably, these limits are obtained considering that both parameters may change simultaneously.

Let us now discuss the importance of keeping a constant optical detuning when performing such optomechanical resonant sensing experiments. It is well-known that, to a greater or lesser extent, optomechanical effects such as radiation pressure or photothermal couplings are always present in optomechanical resonators. These effects induce changes in the device’s mechanical resonant frequencies, whose amplitude depends on many factors such as the injected optical power, the optical and mechanical quality factor, the coupling rates, or the thermal dissipation, among others. Notably, they also depend on the optical detuning between the laser and the optical resonant wavelength of the device Researchers have taken advantage of these effects in order to significantly enhance the device response, which can improve their detection limit.^20,24^ However, using this approach, the mechanical signal acquires a less direct relation to the sensed external perturbation, which may reduce the sensor reliability. Note that in the above experiments, we kept the optical detuning constant. What would have happened if we had not done so?

**Figure 5:**
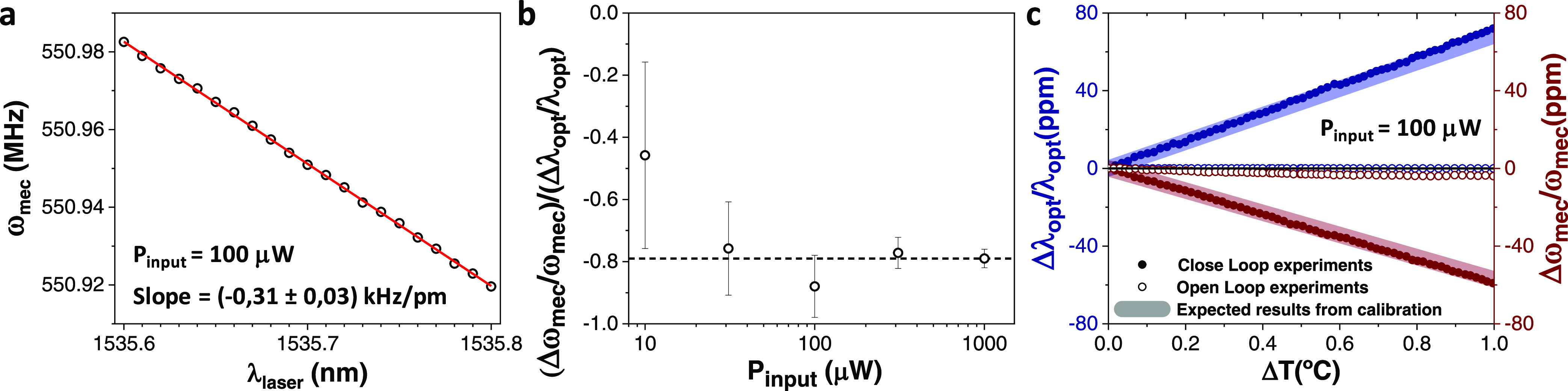
Comparison between open loop and close loop operation modes. (a) Mechanical resonant frequency of the 1st RBM of the disk shown in Figure 1, as a function of the laser optical wavelength (*P*_input_ = 100 μW). Black dots represent the experimental data, while the red line is a linear fit. (b) Ratio of the normalized mechanical frequency shift and the laser wavelength change as a function of the optical input power. (c) Normalized laser optical wavelength change (blue) and mechanical resonant frequency shift (brown) as a function of temperature variation (*P*_input_ = 100 μW), when keeping the laser optical wavelength fixed (open circles) and when actively tracking the optical WGM resonance (full circles). Semitransparent solid lines represent the expected response from previous calibrations (Figure 3c,d).

To answer this question, let us first study the influence of optical detuning on the mechanical resonance of our particular devices. Figure 5a plots the first RBM resonant frequency of a disk as a function of the laser’s optical wavelength. As in previous measurements, the laser optical wavelength is set on the blue flank of the *TE*_1,25,1_ WGM, using here an optical input power of 100 μW. The graph shows that the mechanical frequency decreases linearly with optical wavelength, presenting a slope of (−0.31 ± 0.03) kHz/pm. Note that, due to thermo-optic effects, the analyzed wavelength range is much larger than the WGM full width at half-maximum (Supporting Information Section S2). Considering the previously measured  and , we can prove that the induced shift is dominated by thermal heating. We characterized this dependence for different optical input powers. Figure 5b shows the ratio of the normalized mechanical frequency shift and the laser wavelength change as a function of the optical input power. Apparently, the ratio barely depends on the optical input power, even if at a low optical input power (10 μW) we have a larger uncertainty in the measurement. Note that in the analyzed optical power range, optomechanical effects do not significantly amplify or cool the mechanical modes, thus, our experiments lie on the Brownian motion regime (Supporting Information Section S3).

Lastly, we performed two different experiments monitoring the disk optical and mechanical signals while finely varying the temperature. In the first experiment, we kept the laser optical wavelength fixed. In the second, we maintained the optical detuning constant by activating a feedback loop acting on the laser optical wavelength to maintain the transmitted optical power. Figure 5c plots the normalized laser optical wavelength and RBM resonance frequency as a function of temperature for both experiments. The graph also contains the expected responses from previous calibrations (Figure 3). In order to optimize the optical and mechanical sensitivities, we set the optical input power to 100 μW. Similar results may be obtained for the input power range considered in this work. Obviously, when fixing the laser optical wavelength, information about optical resonant wavelength variations is less direct. To a certain extent, as long as the laser light remains on the blue flank of the WGM resonance, we can access this information by measuring changes in the optical transmission (Supporting Information Section S4), but this approach requires prior characterization of the optical mode. In contrast, actively tracking the WGM resonance provides a direct, real-time measurement of the optical shift. On the mechanical side, the impact of not keeping the optical detuning constant is even more important. In that case, the measured mechanical response is very far from what we expected from calibration. As for the optical case, we can recover the correct information by performing prior characterization experiments (Supporting Information Section S4). In this case, the optical detuning influence on the mechanical resonant frequency was characterized (Figure 5). But this approach is much more tedious than in the optical case, and even more in the case of simultaneous monitoring of several mechanical modes, which is required in many biological/chemical sensing applications. On the other hand, by keeping the optical detuning constant, we retrieved the response from calibrations with very high precision. The residual differences stem from misalignments of the microlensed fibers to the integrated waveguides. It is also important to note that not fixing the optical detuning may decrease the mechanical sensitivity during the experiment, as the optical detuning will not be kept at the optimized value. Moreover, at some point in a continuous sensing experiment, the mechanical and optical signals may be lost, as the laser would drift away from the WGM blue flank. By actively tracking the optical mode, the sensing range is no longer limited.

## Conclusions

In this work, we present the advantages of integrating mechanical and optical resonances in a single sensing platform and introduce the operating principles of such sensors. In particular, we highlight the potential of nano-optomechanical disks, even if our conclusions concern many optomechanical resonators. The proposed devices simultaneously support very high quality optical and mechanical modes confined in tiny volumes, which provide them with uncommon assets for sensing. First, we study their responsivities to relative humidity and temperature changes. Then, we prove that the dual optical/mechanical sensing approach allows disentangling both changes with a very high resolution. Concretely, it enables the detection of variations down to 1 mK in temperature and 0.1% in relative humidity, even in situations where both parameters change at the same time. Finally, we highlight the importance of keeping the optical detuning constant when running this kind of sensors. We demonstrate that actively tracking the optical mode is essential to obtain optical and mechanical signals that are directly related to the external perturbation to be sensed. In addition, it enables widening of the sensing range and avoids degrading the device sensitivity along the experiment. We stress that the conclusions of this work are valid for most sensing applications of optomechanical resonators, including chemical and biological sensing. Implementing sensors that collect multiparametric information from the analytes may be essential to increase their reliability, moreover considering that we are entering into the era of big data science. In this regard, optomechanical resonators present natural assets that are ready to be employed.

## Supporting Information Available

The document provides details about the calculation of the amount of water adsorbed on the sensors during relative humidity experiments, the thermo-optic effects present in our devices, the demonstration of operating in the Brownian motion regime, and the calculation of optical and mechanical signals directly related to the external perturbation when operating in the open loop mode (PDF)
