# Supplementary material for: Simultaneous Optical and Mechanical Sensing Based on Optomechanical Resonators
Source: ACS Sens. 2023 Dec 29;9(1):371–8. doi: 10.1021/acssensors.3c02103 (PMC10825865; doi:10.1021/acssensors.3c02103)
Supplement: Supplementary file 1 — se3c02103_si_001.pdf [file se3c02103_si_001.pdf]

Supporting information

Simultaneous optical and mechanical sensing based on optomechanical resonators

Elena Sentre Arribas,[1] Alicia Aparicio Millán,[1] Aristide Lemaître,[2] Ivan Favero,[3] Javier Tamayo,[4] Montserrat Calleja,[4] and Eduardo Gil-Santos[1*]

[1] Optomechanical Sensors Lab, Instituto de Micro y Nanotecnología, IMN-CNM (CSIC), Isaac Newton 8 (PTM), E-28760 Tres Cantos, Madrid, Spain.

[2] Centre de Nanosciences et de Nanotechnologies, Université Paris-Saclay, CNRS, UMR 9001, 91120 Palaiseau, France.

[3] Matériaux et Phénomènes Quantiques, Université Paris Cité, CNRS, UMR 7162, 75013 Paris, France.

[4] Bionanomechanics Lab, Instituto de Micro y Nanotecnología, IMN-CNM (CSIC), Isaac Newton 8 (PTM), E-28760 Tres Cantos, Madrid, Spain.

[*]Corresponding author: eduardo.gil@csic.es

Section S1: Amount of water adsorption along relative humidity experiments

To estimate the amount of water that is adsorbed on the sensors when varying the relative humidity, we rely on finite element method (FEM) simulations using the commercial software Comsol Multiphysics. Figure S1 show the relative resonant wavelength shift of the Whispering Gallery Mode (WGM) and the relative resonant frequency shift of the Radial Breathing Mode (RBM) of the nano-optomechanical disk (Figure 1) as a function of the number of water layers adsorbed on it. In our simulations, the water layers cover the whole surface of the disk. For the optical and mechanical properties of water, we have used the ones of ice, as it is well known that the first water monolayers adsorbed on surfaces show an iced-like behaviour.[1] The specific values used were: $n=1.291$, $k=5\cdot10^{-3}$, $\rho=917\ Kg/m^3$, $E= 6\ GPa$ and $\upsilon=0.33$;[2] being n the optical refractive index, k the optical adsorption coefficient, ρ the density, E the Young´s modulus and υ the Poisson coefficient. Both optical and mechanical responses show a remarkable linear behavior. Optics wise, the adsorption of water molecules on the disk surface shifts the WGM resonance towards larger wavelengths, with a slope of 27.2 ppm/monolayer. As explained in the main text, this behavior is mainly associated to the increment of the effective refractive index of the WGM (Eq 1). Mechanics wise, water adsorption shifts the RBM resonance towards lower frequencies with a slope of -89.7 ppm/monolayer. As stated in the main text, this response comes from the increment of both, the effective mass and the effective stiffness of the RBM, being the former the dominant one. Experimentally, along increasing the relative humidity from 20% to 45%, the total optical resonant wavelength and mechanical resonant frequency shifts measured were (11.24 ± 0.65) ppm and (-35.8 ±1.8) ppm, respectively. Therefore, from the optical data, we estimate that (41.3 ± 2.4)% of a monolayer got adsorbed during the experiment, while from the mechanical one, we estimate it in (40.0 ± 2.0)%. Notably, both results are in excellent agreement, which validates them. Their average value is (40.6 ± 2.2)%.

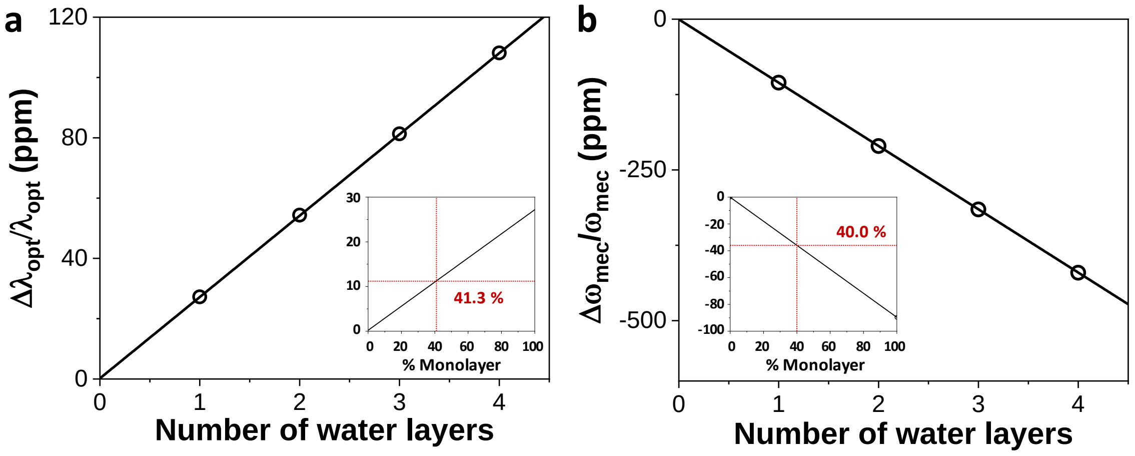

Figure S1. *Modeling the optical and mechanical responsivities to water adsorption. (a) Relative optical resonant wavelength shift as a function of the number of water layers adsorbed on the disk. The inset shows a zoom of the 1st monolayer, plotting also the experimentally measured optical wavelength shift and the calculated percentage of water monolayer adsorbed, when increasing the relative humidity from 20% to 45% (dashed red lines). (b) Relative mechanical resonant frequency shift as a function of the number of water layers adsorbed on the disk. The inset shows a zoom of the 1st monolayer, plotting also the experimentally measured mechanical frequency shift and the calculated percentage of water monolayer adsorbed, when increasing the relative humidity from 20% to 45% (dashed red lines).*

Section S2: Thermo-optic effects

Figure S2 shows the optical spectra of the nano-optomechanical disk acquired at different optical input powers. As the optical input power is increased, the thermo-optic effect, which shifts the optical resonance towards larger wavelengths, becomes increasingly prominent. The thermo-optic effect results from the heating caused by the adsorption of photons in the disk. Photons are adsorbed due to the presence of defects in the disk surface and through two-photon absorption processes. Heating increases the effective refractive index of the disk, thereby shifting its WGM resonance towards higher wavelengths. It is noteworthy that the thermo-optic effect is almost insignificant for the lowest optical input power used (10 μW). In this regime, we estimate that the induced heating is less than 200 mK.

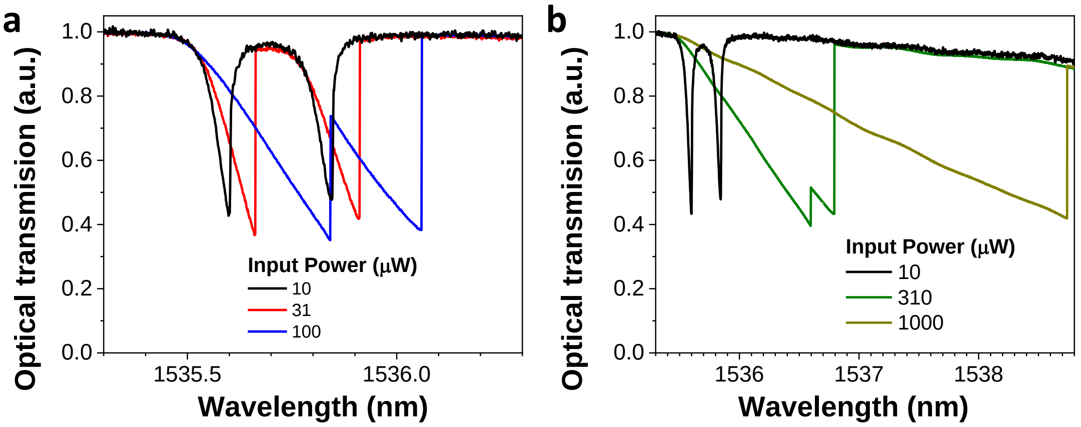

Figure S2. *Optical spectra of the nano-optomechanical disk for different optical input powers. (a) Optical input powers of 10 μW (black), 31 μW (red) and 100 μW (blue). (b) Optical input power of 10 μW (black), 310 μW (green) and 1 mW (dark yellow).*

Section S3: Brownian motion regime

In this section, we analyze the impact of optomechanical effects on the dynamics of the nano-optomechanical disk 1st RBM, with the aim of determining whether our experiments lie on the Brownian motion regime. Figure S3a-b show the normalized mechanical spectra of the nano-optomechanical disk acquired at different optical input powers. Note that the laser was set at a wavelength that maintained the normalized optical transmission, in order to keep the optical detuning constant throughout all measurements. For shake of clarity, Figure S3c shows the evolution of the mechanical resonant frequency and the mechanical quality factor as a function of the optical input power. Regarding the mechanical resonant frequency, it shifts towards lower values as the optical input power increases. This effect come from both, the presence of optomechanical forces and thermal heating, being the latter the dominant effect. As for the previously analyzed thermo-optic effect, heating comes from the adsorption of photons in the disk. Regarding the mechanical quality factor, it continuously increases as the optical input power rises. This behavior indicates that optomechanical forces alter the dynamics of the RBM. However, the increment is not very high. In particular, at an optical input power of 100 µW, the optical quality factor increases less than 1.5%, respect to the intrinsic one. Note that this is the optical input power that provides better optical resonant wavelength and mechanical resonant frequency stabilities, as well as the one used for comparing the open loop and close loop operation modes in the main text. In addition, at an optical input power of 310 µW and 1 mW, the optical quality factor increments are lower than 3% and 15%, respectively. This data confirms that our experiments mostly lie in the Brownian motion regime.

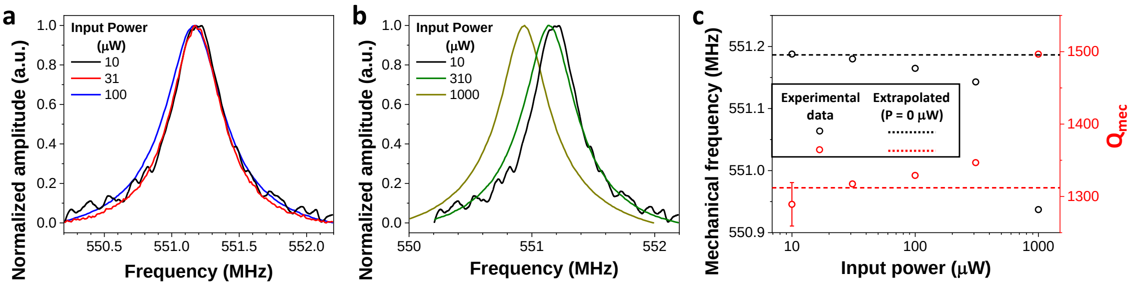

Figure S3. *Dynamics of the nano-optomechanical disk 1st RBM for different optical input power. (a) Mechanical spectra for optical input powers of 10 µW (black), 31 µW (red) and 100 µW (blue). (b) Mechanical spectra for optical input powers of 10 µW (black), 310 µW (green) and 1 mW (dark yellow). (c) Mechanical resonant frequency (black) and mechanical quality factor (red) as a function of the optical input power. The dashed lines indicate the intrinsic mechanical resonant frequency (black) and the intrinsic mechanical quality factor (red).*

Section S4: Calculating optical and mechanical signals directly related with the external perturbation from open loop configuration experiments

As stated in the main text, when using optomechanical resonators for sensing, actively tracking the optical mode is essential to obtain optical and mechanical signals that are directly related with the external perturbation. Not doing so requires prior calibration of the devices. Whereas the optical calibration is simple, the mechanical one is quite tedious. In addition, not actively tracking the optical mode significantly reduces the sensing range and may alter the optical and the mechanical sensitivities during the

experiment, which influences the measurement reliability. In this section, we show how to retrieve mechanical and optical signals that are directly related with the external perturbation, when performing sensing experiments in open loop configuration, (not actively tracking the optical mode). In particular, we exhibit it based on the results obtained along the last experiment shown in the main text, in which we have finely tune the device temperature. For this experiment, we used an optical input power of 100 μW. Figure S4a shows the device optical spectra at this optical input power, indicating the operation point when starting the experiment (λ_{laser}*=1535.8 nm* and *Trans=0.125 mV*; where *Trans* refers to the optical transmission). Note that, the optical transmission around this point can be expressed as: Trans= $860.173\, mV - 0.56\, \frac{mV}{nm} \cdot \lambda_{laser}$ (blue line in Figure S4a). In the main text we shown that, in the open loop configuration, the optical and the mechanical signals do not correspond at all with the expected ones (Figure 5c). Obviously, in this configuration, we cannot get optical information from the variation of the laser wavelength because it is fixed. However, we can get it from the optical transmission changes. Figure S4b shows that the optical transmission increases as the temperature rises, which indicates that the optical resonant wavelength shifts towards larger wavelengths. In order to retrieve the optical wavelength evolution from the optical transmission one, we just need to consider the dependence obtained through the fitting shown in figure S4a, finally getting:

$$\frac{\Delta\lambda_{opt}}{\lambda_{opt}} = 1.16 \cdot 10^{-3} \cdot mV^{-1} \cdot \Delta Trans \qquad \text{(S1)}$$

Figure S4c shows the calculated optical wavelength shift (blue solid circles) as a function of temperature variation, together with the expected response (semitransparent blue line). Notably, they show an excellent agreement. Once calculated the optical wavelength shift evolution, the mechanical one can also be retrieved. For this purpose, we have to take into account that the measured mechanical frequency shift comes from the addition of two effects. On the one hand, the mechanical frequency shift induced by the temperature changes and, on the other hand, the mechanical frequency shift induced by the optical detuning change, which also arises from temperature variation. Therefore, we get:

$$\left(\frac{\Delta\omega_{mec}}{\omega_{mec}}\right)_{measured} = \left(\frac{\Delta\omega_{mec}}{\omega_{mec}}\right)_{original} + \left(\frac{\Delta\omega_{mec}}{\omega_{mec}}\right)_{optical\ detuning} \qquad \text{(S2)}$$

$being\ \left(\frac{\Delta\omega_{mec}}{\omega_{mec}}\right)_{optical\ detuning} = \mathcal{R} \cdot \left(\frac{\Delta\lambda_{opt}}{\lambda_{opt}}\right)_{calculated}$, where $\mathcal{R} = \frac{\Delta\omega_{mec}}{\omega_{mec}} / \frac{\Delta\lambda_{opt}}{\lambda_{opt}}$. Note that, in the main text, we already calculated $\mathcal{R}$ for different optical input powers (Figure 5a-b). We concluded that, for our devices and the particular analyzed range, $\mathcal{R}$ does not depend on the optical input power, taking the value: $\mathcal{R} = -0.81 \pm 0.03$. Considering it and using eq S2, together with the measured mechanical frequency shift (red open circles in Figure 5c) and the calculated optical wavelength shift (blue solid circles in Figure S4c), we can retrieve the mechanical frequency shift that is directly related with temperature changes. Figure S4c plots it (brown solid circles) together with the expected response (semitransparent brown line). As for the optics, they show an excellent agreement. Note again that this kind of treatment can only be performed for a limited range, regarding the optical wavelength change, because at some point optical and mechanical changes may

become nonlinear. Moreover, if the optical wavelength change is large enough, the laser wavelength may be completely out of the WGM flank, definitely losing both signals.

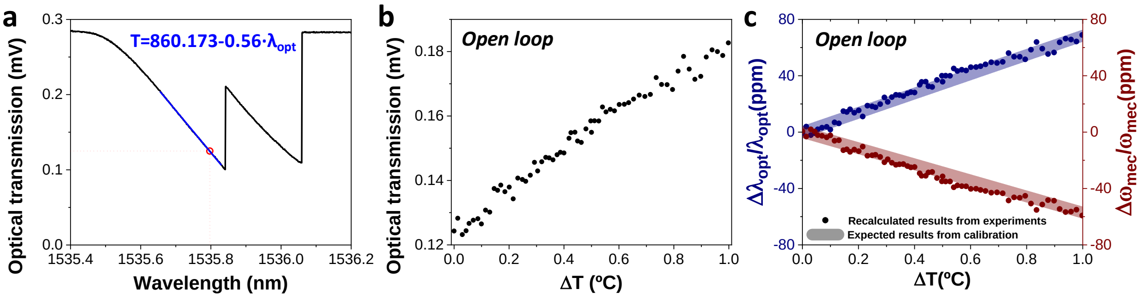

Figure S4. *Calculating optical and mechanical signals that are directly related with the external perturbation, when performing experiments in open loop configuration. (a) Optical spectra of the nano-optomechanical disk using an optical input power of 100 μW. The graph shows the operation point when starting the experiment (red dashed lines). It also show a linear fit (blue line) around that point. (b) Optical transmission of the device as a function of temperature variation. (c) Normalized optical wavelength change (blue solid circles) and mechanical resonant frequency shift (brown solid circles) as a function of temperature variation. Semi-transparent solid lines represent the expected response from previous calibrations (Figures 3c-d).*

REFERENCES

1. Asay, D. B.; Kim, S. H. Evolution of the adsorbed water layer structure on silicon oxide at room temperature. *J. Phys. Chem. B* **2005,** *109*, 16760-16763.
2. Xu, D.; Liechti, K. M.; Ravi-Chandar, K. Mechanical probing of icelike water monolayers. *Langmuir* **2009,** *25*, 12870-12873.
